# Metanephric adenoma: molecular study and review of the literature

**DOI:** 10.18632/oncotarget.28192

**Published:** 2022-02-17

**Authors:** Enrique Rodríguez-Zarco, Jesús Machuca-Aguado, Laura Macías-García, Ana Vallejo-Benítez, Juan José Ríos-Martín

**Affiliations:** ^1^Pathology Department, University Hospital Virgen Macarena, Seville, Spain; ^2^School of Medicine, University of Seville, Seville, Spain; ^3^Pathology Department, Regional University Hospital of Malaga, Malaga, Spain

**Keywords:** metanephric adenoma, BRAF, renal neoplasms

## Abstract

Introduction: Metanephric adenoma (MA) is an uncommon benign tumor accounting for 0.2–0.7% of adult renal epithelial neoplasms. The clinical course is often indolent, but diagnosis should not be delayed since clinical symptoms (hematuria, fever, palpable abdominal mass, and flank pain) may be non-specific and overlap with those of a malign renal neoplasm. We report on 4 cases of AM, for which morphological and mutational analysis were performed.

Material and Methods: Immunohistochemical staining was performed on sections cut from paraffin blocks to assess expression of WT1, vimentin, racemase, CK7, CD10 and RCC. Testing for the BRAF gene mutation V600 was carried out using real-time PCR (Cobas^®^ 4800).

Results: In all four cases, tumors were visible as well-circumscribed, non-encapsulated masses located in the renal cortex and extending towards the medulla. At immunohistochemical examination, tumor cells stained negative for CK7, CD10 and RCC and positive for both WT1 (nuclear, intense) and vimentin (cytoplasmic, intense, and diffuse). Molecular analysis revealed the BRAF gene mutation V600E in three cases and wild-type BRAF in the fourth.

Conclusions: BRAF molecular mutation analysis may aid diagnosis in cases with atypical histological features, especially in small incisional biopsies when reassessment of surgical treatment may be considered.

## INTRODUCTION

Metanephric adenoma (MA) is a rare benign tumor of the kidney, accounting for 0.2% of adult renal epithelial neoplasms [1]. The tumor, which is composed of primitive metanephric cells [2], is often asymptomatic. Some authors have suggested that MA may derive from maturing nephroblastomas (Wilms tumor), since immunophenotypic findings overlap closely with those of differentiated nephroblastoma and nephrogenic rests [3]. Though it may also occur in children, it is detected mainly in adults aged between 50 and 70 and is more common (ratio 2:1) in women [2, 4, 5]. Radical nephrectomy, cryoablation and radiofrequency have been used to treat this neoplasm [5].

While the clinical course is benign, histological findings often overlap with those of malignant tumors including Wilms tumor and renal papillary neoplasms, thus prompting the need for differential diagnosis [6]. A better understanding of this benign tumor would undoubtedly aid the development of less invasive strategies. Although most authors rule out the possibility of MA becoming malignant, one case has been reported of a metanephric adenoma in association with a high-grade sarcoma (metanephric adenosarcoma) [5, 7].

Immunohistochemical analysis is a useful tool for differential diagnosis. The literature contains few reports of integrated diagnosis of MA using molecular techniques [8].

Mutation of the BRAF gene prompts constitutive activation of the ERK-mediated signaling pathway, favoring cell proliferation and differentiation.

Activation of RAF, in both its homodimer and heterodimer forms, triggers the phosphorylation of MAPK kinase (MEK), which in turns prompts the phosphorylation of extracellular signal-regulated kinase (ERK); ERK activation promotes cell proliferation and signal transformation through interaction with several molecules crucial to tumor pathogenesis [9, 10].

The specific BRAF gene mutation V600E has been reported in over half of all cutaneous melanomas and papillary thyroid carcinomas, as well as in a number of blood cancers including hairy cell leukemia; it is also present in indolent and benign tumors such as melanocytic nevus [11]. This specific mutation has also been studied in malignant neoplasms such as renal cell carcinoma in response to targeted therapies [12]. In benign neoplasms like MA, several authors have noted that testing for the BRAF V600E mutation may be a valuable tool for the diagnosis [1, 2, 11].

## RESULTS

**Figure 1:**
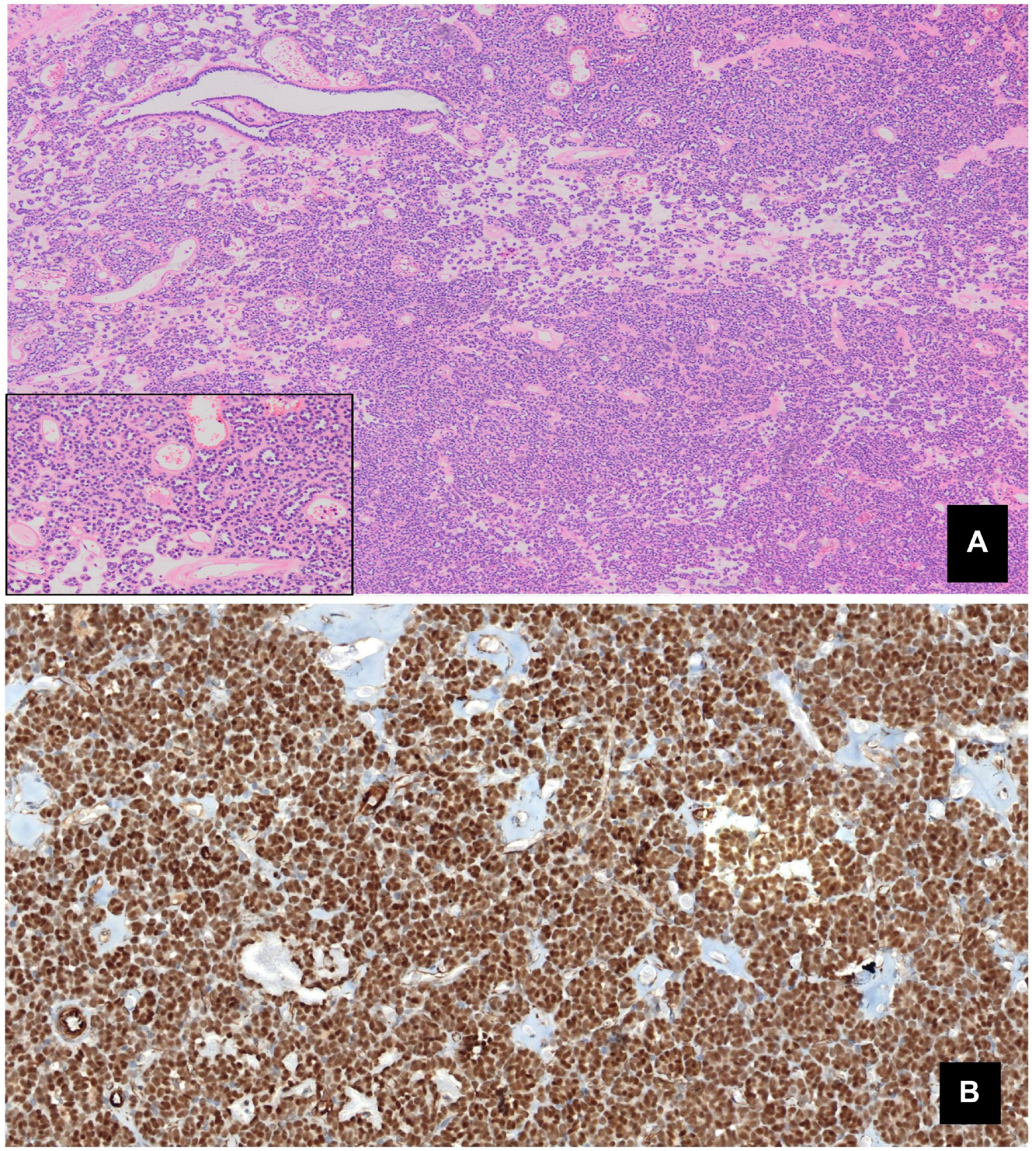
(**A**) Monomorphic epithelial proliferation, displaying no significant atypia or mitotic figures, within an edematous stroma containing psammoma bodies (Panoramic view. HE). (**B**) Intense positive nuclear staining for WT1.

Gross examination revealed well-circumscribed, non-encapsulated tumors measuring between 1.5 and 6 cm (mean 3.7 cm), with focal areas of blood-containing cysts and solid, in some cases presenting calcifications. Histologically, MAs were composed of small, monomorphic epithelial cells displaying no significant atypia or mitotic figures, showing papillary or acinar patterns, and edematous or hyalinized stroma with calcification (psammoma bodies) (Figure 1A). Immunohistochemical examination showed positive staining for WT1 and vimentin, and negative staining for racemase, CK7, CD10, CD57 and RCC (Figure 1B). Mutation analysis by real-time PCR revealed the BRAF gene mutation V600E in three cases and wild-type BRAF in the fourth. Based on morphological features and the findings of immunohistochemical and molecular analysis, all four cases were diagnosed as MA. Three of the patients are alive and well 12, 5 and 2 years after surgery, while the fourth died 13 years after the procedure due to other causes.

## DISCUSSION

MA is an uncommon, benign tumor of the kidney composed of spindle cells associated with epithelial cells. In 1988, Mostofi et al. [13] described MA for the first time as a distinct nosologic entity among renal neoplasms, with tubular-like epithelial cells. This tumor is currently classified among the metanephric neoplasms, which also include metanephric adenoma and metanephric stromal tumour [4]. MA, which accounts for 0.2–0.7% of adult renal epithelial neoplasms, derives from remnants of embryonic renal tissue [1, 3, 4].

At gross examination, MA appears as a well-circumscribed neoplasm with a yellowish surface, often displaying evidence of secondary changes including focal necrosis, hemorrhage and/or cystic degeneration. Coarse calcification may also be present. The tumor mass generally ranges in size between 3 and 6 cm in diameter, although tumors of up to 15 cm have been reported [4]. Histologically, it comprises an acinar arrangement of small cells. Differential diagnosis of MA includes Wilms tumor, nephrogenic rests and renal papillary neoplasms [5]. MA typically expresses WT1 and CD57, but stains negative for CK7 and racemase. Positive intense staining for WT1 was recorded in the four cases reported here, but CD57 staining was in most of them weak; so, BRAF mutation helped us to confirm the diagnosis of MA.

Oncogenic BRAF normally regulates cell division and differentiation through the MAP kinase signaling pathway. BRAF mutations, identified in several solid tumors and blood cancers, prompt the constitutive activation of the pathway, which has been widely documented in melanomas [9, 10, 14]. Most BRAF mutations involve a thymine-adenine transversion, leading to the substitution of valine by glutamic acid at codon V600 (V600E) [14].

Most reported MAs display a normal genotype, lacking the simultaneous chromosomes 7 and 17 gain and Y chromosome loss characteristic of papillary renal cell carcinoma and common in neuroblastoma.

The BRAF gene mutation V600E is reported in roughly 90% of metanephric adenomas [2, 4, 11, 15], with only 2 described cases of V600D mutation [2] and 1 of V600K [16]. Caliò [14] et al. identified the V600E mutation in 41 out of 48 MA patients (85%) with a mean age of 54, while Choueiri et al. [11] reported it in 26/29 patients (89%) also with a mean age of 54, and Ding et al. [17] described 27 MA patients with mean age of 39 years and 22 (81%) with BRAF mutation. Other authors have reported the V600E mutation in smaller series [18–21]. In our research, as shown in Table 1, the mutation was identified in three of the four patients studied (75%), aged between 19 and 65 (mean 47.5).

**Table 1:** BRAF mutations in metanephric adenomas: literature review

Research and year	Number of cases	Mean age (year, range)	Gender	Tumor size (cm, range)	BRAF mutation	Type of mutation
Previous reports in Caliò et al., 2016	99	52 (5–84)	71F 28M	3.4 (1.1–8)	87 (88%)	V600E (97) V600D (2)
Ding et al., 2018	27	39 (12–80)	9F 18M	3.1 (2–7)	22 (81%)	V600E
Wobker et al., 2019	10	42 (10–62)	6F 4M	2.7 (1.3–3.5)	8 (80%)	V600E
Catic et al., 2020	28	52 (9–73)	17F 10M	3 (0.5–12)	15 (53%)	V600E
Chan et al., 2020	12	54 (38–76)	11F 1M	2.9 (1–6)	12 (100%)	V600E
Lenci et al., 2021	1	73	F	3.2	1 (100%)	V600K
Current study	4	54 (19–65)	1F 3M	3,7 (1.5–6)	3 (75%)	V600E

Table adapted from Caliò et al. [14].

Several authors have drawn attention to the relationship between wild-type BRAF and MA in younger adults (i.e., under 25). Of the 29 cases reported by Choueri [11] et al., all three patients harboring wild-type BRAF were well below the mean age. By contrast, in the four pediatric cases of MA described by Chami et al. [22], only one harbored wild-type BRAF. An epidemiological analysis was carried out of the published cases of MA in which the BRAF mutation has been studied. In Table 2 it can be observed that, although the age range of presentation is similar in both groups, the mean age is 19.6 years lower in the wild-type BRAF patients (31.1 vs. 50.7). The present study detected the BRAF gene mutation V600E in patients aged between 50 and 65, while the youngest patient (aged 19) harbored wild-type BRAF.

**Table 2:** Comparison of age and gender incidence in mutated BRAF and wild type BRAF metanephric adenomas: literature review

Research and year	Number of MUTATED/WT cases	Mean age MUTATED/WT (years, range)	Gender			
			MUTATED BRAF		WT BRAF	
			Male	Female	Male	Female
Choueiri et al., 2012	26/3	54.7 (36−78)/32 (25−38)	3 (12%)	23 (88%)	−	3 (100%)
Dadone et al., 2013	1/0	61/−	−	1 (100%)	−	
Pinto et al., 2015	6/0	52/−	−	6 (100%)	−	
Udager et al., 2015	10/1	51.2 (16−84)/32	4 (40%)	6 (60%)	1 (100%)	−
Chami et al., 2015	3/1	5.6 (4−9)/10	2 (67%)	1 (33%)	1 (100%)	−
Caliò A et al., 2016	41/7	57 (5−84)/33 (10−74)	11 (27%)	30 (73%)	6 (86%)	1 (14%)
Ding et al., 2018	22/5	40 (25−73)/29 (12−47)	17 (77%)	5 (23%)	4 (80%)	1 (20%)
Wobker et al., 2019	8/2	46 (19−62)/26.5 (10−43)	3 (37%)	5 (63%)	1 (50%)	1 (50%)
Catic et al., 2020	15/9	47 (5−75)/36 (9−71)	7 (46%)	8 (54%)	3 (37%)	5 (63%)
Chan et al., 2020	12/0	54 (38−76)/−	1 (8%)	11 (92%)	−	
Lenci et al., 2021	1/0	73/−	−	1 (100%)	−	
Current study	3/1	57 (50−65)/19	2 (67%)	1 (33%)	1 (100%)	−
	148 (84%)/29 (16%)	50.7 (4−84)/31.1 (9−74)	50 (34%)	98 (66%)	17 (58%)	12 (42%)

A relationship that has not been previously highlighted is the higher incidence of wild-type BRAF in male patients. As described above, metanephric adenoma is more common in female patients in a 2: 1 ratio, just like in mutated BRAF MA (Table 2). By contrast, in wild-type BRAF MA, the incidence in men is higher than women with a 1.45: 1 ratio. A remarkable case is the series of 48 patients by Calio et al. [14], where the F:M ratio in mutated BRAF is 2.7: 1, while in wild-type BRAF it is 1: 6.

Therefore, mutated BRAF MA are more frequent in elderly patients and women, which is consistent with studies in other pathologies. Even with these results, it is necessary to carry out studies with a greater number of cases in order to ensure it.

## MATERIALS AND METHODS

This paper reports on MA in three men and one woman, aged between 19 and 65 (mean age 47.5); patient data are provided in Table 3. The diagnosis was confirmed and subsequently reviewed following WHO-recommended criteria [4]. In all cases, MA presented as a single, asymptomatic mass discovered incidentally during imaging procedures; CT scan confirmed the presence of a solitary, space-occupying, solid renal tumor. A nephrectomy was performed in all patients.

**Table 3:** Epidemiological data and BRAF status

Case	Gender	Age (years)	*BRAF* status (V600E)
1	M	19	WT
2	F	50	Mut
3	M	56	Mut
4	M	65	Mut

Abbreviations: M: male; F: female; WT: wild type; Mut: mutated.

Immunohistochemical staining was performed on H&E- stained sections cut from paraffin blocks to assess expression of WT1, vimentin, racemase, CK7, CD10 and RCC. Testing for the BRAF gene mutation V600 was carried out by real-time PCR (Cobas^®^ 4800) using the DNA Sample Preparation Kit and the BRAF Mutation Test (Roche), which detects the BRAF gene mutations V600E, V600K and V600D in formalin-fixed, paraffin-embedded tissue.

## CONCLUSIONS

These results bear out the findings of previous studies of BRAF gene mutations in MA, showing that molecular mutation analysis may aid diagnosis in cases with atypical histological features, especially in small biopsies when non-surgical treatment is planned.

A highly accurate definitive diagnosis of MA was achieved by combining immunohistochemical and molecular analysis; mutated BRAF MA are more frequent in elderly patients and women. Accurate early diagnosis may help to avoid unnecessary aggressive treatments such as radical nephrectomy.
